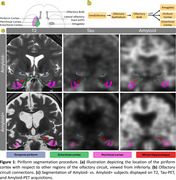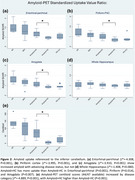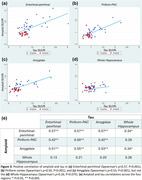# Quantification of Amyloid and Tau associations in the Piriform Cortex in Alzheimer's and Parkinson's Disease using PET‐MR

**DOI:** 10.1002/alz70856_096159

**Published:** 2025-12-24

**Authors:** Hossein Moein Taghavi, Mahta Karimpoor, Eric van Staalduinen, Christina B. Young, Marios Georgiadis, Mackenzie L. Carlson, America Romero, Alexandra N. Trelle, Hillary Vossler, Maya Yutsis, Jarrett Rosenberg, Guido A. Davidzon, Greg Zaharchuk, Kathleen L. Poston, Anthony D. Wagner, Victor W. Henderson, Elizabeth C. Mormino, Michael Zeineh

**Affiliations:** ^1^ Stanford University School of Medicine, Stanford, CA, USA

## Abstract

**Background:**

Olfactory‐associated regions, particularly the piriform cortex, may harbor early pathological changes in Alzheimer's (AD) and Parkinson's disease (PD). Amyloid–tau overlap is strongest in the medial temporal lobe. Amyloid–tau association in the piriform cortex versus other medial temporal structures remains unclear. We used dual PET‐MR to quantify simultaneous amyloid/tau uptake in the piriform cortex, entorhinal–perirhinal cortices, amygdala, and hippocampus among AD, mild cognitive impairment (MCI), amyloid‐positive/negative healthy controls (HC), and PD cohorts.

**Method:**

We analyzed PET‐MR cross‐sectional data (*n* = 47): 7 AD, 8 MCI, 13 amyloid+HC, 8 amyloid‐HC, and 11 PD enrolled in Stanford's ADRC and SAMS studies. Amyloid ^18^F‐Florbetaben and tau‐PET ^18^F‐PI‐2620 tracers were used, including sagittal T1‐weighted and coronal T2‐weighted fast‐spin‐echo MRI. Automated segmentation of entorhinal–perirhinal cortices and the whole hippocampus= (CA1–4+dentate gyrus+subiculum) was performed. Three sub‐regions of the piriform cortex were manually segmented blind to diagnosis: frontal, temporal, and the contiguous periamygdaloid‐cortex, Figure 1.

**Result:**

*Amyloid SUVr (Figure 2)*:

Global amyloid uptake was positively associated with ordinal disease category (whole‐brain centiloid scores: non‐parametric Jonckheere‐Terpstra test J*=‐4.89, *p* <0.001), including the regional amyloid in entorhinal‐perirhinal (J*=‐4.31, *p* <0.001), piriform‐PAC (J*=‐3.40, *p* <0.001), and amygdala (J*=‐3.91, *p* <0.001), but not in the whole hippocampus (J*=‐1.41, *p* = 0.080).

Rank‐sum tests showed that amyloid+HC had higher global amyloid burden than amyloid‐HC (centiloid scores: z=3.77, *p* <0.001). In the medial temporal lobe, this included elevated amyloid in piriform‐PAC (z=‐2.46, *p* = 0.014), entorhinal‐perirhinal (z=‐3.26, *p* = 0.001), and amygdala (z=‐2.58, *p* = 0.007), but not in whole hippocampus (z=‐1.23, *p* = 0.218).

There were no differences between PD and amyloid‐HC global amyloid uptake (centiloid scores: z=‐1.64, *p* <0.001), including regional amyloid in entorhinal‐perirhinal (z=‐0.41, *p* = 0.680), piriform‐PAC (z=‐0.33, *p* = 0.741), amygdala (z=‐0.58, *p* = 0.563), and whole hippocampus (z=‐0.83, *p* = 0.409).

*Amyloid/Tau Correlations (Figure 3)*:

Amyloid was positively correlated with tau uptake in the entorhinal‐perirhinal (Spearman's *ρ* = 0.57, *p* <0.001), piriform‐PAC (Spearman's *ρ* = 0.50, *p* <0.001), and amygdala (Spearman's *ρ* = 0.53, *p* <0.001) but not the whole hippocampus (Spearman's *ρ* = 0.26, *p* = 0.075). Amyloid within entorhinal‐perirhinal, piriform‐PAC, and amygdala was correlated with tau in most regions, excluding the whole hippocampus.

**Conclusion:**

We show early increases in primary olfactory cortex amyloid‐PET, correlating strongly with tau uptake, similar to entorhinal cortex and amygdala, but distinct from hippocampus.